# Rationale and Clinical Research Progress on PD-1/PD-L1-Based Immunotherapy for Metastatic Triple-Negative Breast Cancer

**DOI:** 10.3390/ijms23168878

**Published:** 2022-08-10

**Authors:** Yifan Ren, Jialong Song, Xinyi Li, Na Luo

**Affiliations:** Department of Histology, School of Medicine, Nankai University, Tianjin 300071, China

**Keywords:** cancer therapy, metastatic triple-negative breast cancer, anticancer drugs, immunotherapy, immune checkpoint blockade therapy, PD-1/PD-L1, clinical trails

## Abstract

Metastatic triple-negative breast cancer (mTNBC), a highly aggressive and malignant tumor, currently lacks an effective treatment. There has been some progress in the treatment of mTNBC with programmed death receptor-1/programmed death ligand-1 (PD-1/PD-L1) immunotherapy in recent years. The combination of PD-1/PD-L1 inhibitors with other therapies is a noteworthy treatment strategy. Immunotherapy in combination with chemotherapy or small-molecule inhibitors still faces many challenges. Additionally, there are some new immunotherapy targets in development. We aimed to further evaluate the effectiveness and usefulness of immunotherapy for treating mTNBC and to propose new immunotherapy strategies. This review explains the rationale and results of existing clinical trials evaluating PD-1/PD-L1 inhibitors alone or in combination for the treatment of mTNBC. For patients with aggressive tumors and poor health, PD-1/PD-L1 inhibitors, either alone or in combination with other modalities, have proven to be effective. However, more research is needed to explore more effective immunotherapy regimens that will lead to new breakthroughs in the treatment of mTNBC.

## 1. Introduction

The impact of malignant tumors on human health is increasing with changes in human habits and the average life expectancy. One study showed that the morbidity and mortality rates of breast cancer have significantly increased worldwide over the past two decades, and as of today, breast cancer has become the leading cause of death for women worldwide [1].

Triple-negative breast cancer (TNBC) refers to a type of breast cancer in which the immunohistochemistry of the cancer tissue is negative for estrogen receptor (ER), progesterone receptor (PR), and human epidermal growth factor receptor-2 (HER-2), and it accounts for 15–20% of all breast cancer patients [2]. Because of its rapid progression, most patients with TNBC have progressed to the more malignant and aggressive metastatic TNBC (mTNBC), with a shorter survival period by the time they seek medical attention. The majority of breast cancer deaths are caused by mTNBC. According to pathological characteristics, it lacks specific therapeutic targets, and it cannot be completely removed surgically due to unclear distant micro-metastases. Therefore, treatment of mTNBC is usually based on chemotherapy. However, according to clinical statistics, the overall response rate (ORR‘) of mTNBC with single-agent chemotherapy is only 10–30%, and with the best multi-drug combination chemotherapy regimen it is only 63%. The average pathologically complete response (pCR) to mTNBC with multi-drug combination chemotherapy regimen is about 30–40% [3]. In summary, the benefit of chemotherapy for patients with mTNBC is not promising [4,5]. The search for treatments with high clearance, good targeting, and few side effects has become a major focus of medical research.

In recent years, immunotherapy has become an important development direction for future tumor treatment; is known as the fourth treatment modality after surgery, radiotherapy, and chemotherapy; and is considered one of the most popular therapies used to treat tumors [6]. Among all types of breast cancer, TNBC is the most immunogenic; therefore, an increasing number of immune-related studies have been conducted at home and abroad to explore therapeutic mTNBC approaches, the most important of which are those targeting the PD-1/PD-L1 pathway. These are characterized by higher levels of PD-1 and PD-L1 expression, with a clear correlation with high expression of tumor infiltrating lymphocytes (TILs) [7]. The results of several studies also show that patients with TNBC who have more TILs and higher PD-L1 expression levels have better prognosis [8,9,10]. This suggests that immunological approaches to the treatment of mTNBC are highly effective and will become an important pathway.

In this study, we analyzed and discussed the progress in immunotherapy related to PD-1/PD-L1 inhibitors for mTNBC.

## 2. Immune Checkpoint and Immune Checkpoint Blockade Therapy

**Figure 1:**
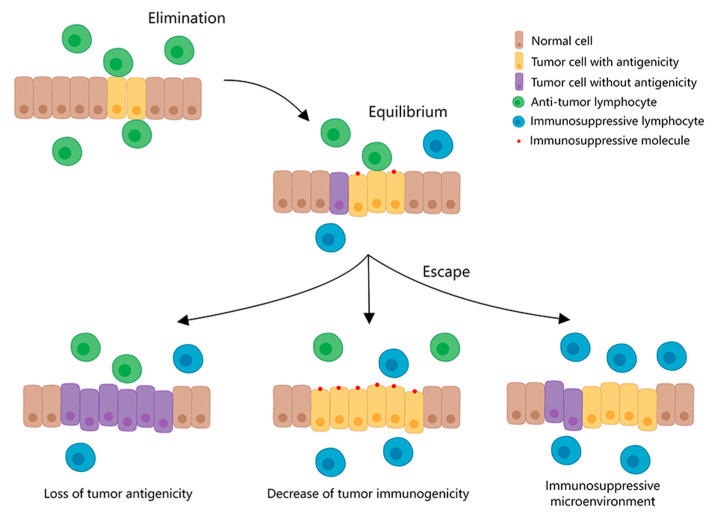
Immune escape mechanism of tumors. Along with tumor growth, the immune system develops immune tolerance to tumor cells due to weakened antigenicity of tumor cells, reduced responsiveness to immune killing mechanisms, and expression of immunosuppressive molecules.

Under normal circumstances, the human immune system functions in immune surveillance and elimination, but as the tumor grows, the tumor cells develop immune-suppressive responses, such as weakened antigenicity of the tumor cells, reduced responsiveness to the immune killing mechanism, and expression of immunosuppressive molecules. Under these circumstances, the immune system develops immune tolerance to tumor cells, known as immune editing (Figure 1) [11,12]. Immunotherapy for tumors is based on immune editing, applying immunological principles and methods to reactivate immune cells, enhance the anti-tumor immune response, break the immune tolerance, and inhibit tumor growth by enhancing the antigenicity of tumor cells and the killing ability of immune cells, and inhibiting the effect of immunosuppressive molecules. It mainly includes immune checkpoint blocking therapy [13], therapeutic antibodies [14], cancer vaccines [15], adoptive cellular immunotherapy [16], small-molecule inhibitors [17], and other methods. The common immunotherapy approaches are shown in Table 1.

Immune checkpoint therapy (ICT) blocks the action of immune checkpoints by artificially administering inhibitors of immune checkpoints or their ligands, thereby upregulating T cells activity and improving the anti-tumor immune response. The Food and Drug Administration (FDA) has approved multiple immune-checkpoint-blocking drugs for cancer treatment because of the advantages of this method, such as being highly targeted and not prone to tumor resistance [23]. Currently, PD-1/PD-L1 and CTLA-4/B7-1 are the primary targets of immune checkpoint blockade therapies. In addition, molecules such as lymphocyte activation gene-3 (LAG-3), T-cell immunoglobulin and mucin domain 3 (TIM-3), and T-cell immunoglobulin and ITIM domains (TIGIT) have also been extensively investigated as targets.

PD-1, also known as CD279, is a 55 kDa transmembrane protein. It is mainly expressed by activated T cells, B cells, and natural killer cells and is significantly highly expressed by tumor-specific T cells. PD-L1, also known as CD274 or B7-H1, belongs to the B7 family and is a 33 kDa transmembrane glycoprotein. This protein is normally expressed by macrophages, activated T cells, and B cells, and its expression in tumor cells increases with the progression of the disease and/or with the degree of heterogeneity of tumor cells. When PD-1 is combined with PD-L1, it can inhibit the activation and proliferation of T cells in peripheral tumor tissues and attenuate the cell-killing effect of T cells by regulating the PI3K-AKT-mTOR [24] and Ras-EMK-ERK pathways [25]. In addition, tumor cells can be stimulated to grow and invade, causing immunosuppression, inhibiting the secretion of pro-inflammatory factors, and weakening the antigen-presenting ability of dendritic cells, which leads to the immune escape of tumors [13].

Cytotoxic T lymphocyte-associated antigen-4 (CTLA-4), also known as CD152, is a leukocyte differentiation antigen that functions primarily in the T cell activation phase of lymphoid organs. As a transmembrane receptor on the surface of T cells, CTLA-4 inhibits T cell hyperactivation by competitively binding ligand B7-1/2 (CD80/86) to CD28, the activating receptor of T cells [26].

**Figure 2:**
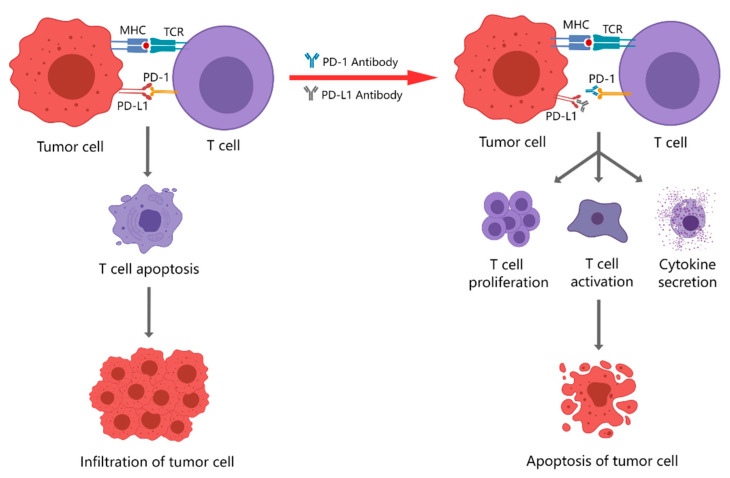
Effect of PD-1 and PD-L1 inhibitors. The combination of PD-1 and PD-L1 induces T-cell apoptosis, so the tumor cells will infiltrate; the use of PD-1 or PD-L1 inhibitors promotes T-cell proliferation, activation and secretion of cytokines, and enhances the tumor-killing effect of T cells.

PD-1/PD-L1 therapies are more specific and act faster because PD-1 acts mainly in peripheral tumor sites and works in the T cell effector phase, while CTLA-4 acts mainly in lymphoid organs and works in the T cell activation phase [27]. Several studies have compared the adverse effects of treatment with CTLA-4 and PD-1/PD-L1 inhibitors and found that CTLA4 inhibitors have more side effects than PD-1/PD-L1 inhibitors [28]. Therefore, immunotherapy with PD-1/PD-L1 inhibitors can enhance anti-tumor immunity, which is more suitable for patients in poor condition and with aggressive tumors and is important for the treatment of rapidly progressing mTNBC (Figure 2).

## 3. PD-1/PD-L1 Inhibitors Currently Used for Clinical Treatment

### 3.1. PD-1 Inhibitors

PD-1 inhibitors are monoclonal antibodies that bind to the PD-1 on T cells, effectively inhibiting the binding of PD-1 to PD-L1 and PD-L2 receptors on cancer cells, allowing the immune escape of tumor cells to be recognized by T cells and exert anti-tumor effects. Studies have shown that PD-1 monoclonal antibodies do not bind Fc or activate complements during the blockade of PD-1; therefore, they are not cytotoxic [29]. Commonly used PD-1 inhibitors are listed in Table 2 [30,31].

### 3.2. PD-L1 Inhibitors

PD-L1 inhibitors are monoclonal antibodies engineered from human PD-L1 that target PD-L1 on tumor cells and inhibit the PD-1/PD-L1 pathway, thereby reactivating anti-tumor immunity. The durable safety and long-term clinical benefits of monoclonal antibodies against PD-L1 have led the FDA to approve them for use in the treatment of many types of cancers (Table 3) [32,33,34]. 

Current PD-1/PD-L1 inhibitor treatment modalities for mTNBC include monotherapy, as well as combination therapy with chemotherapy or small-molecule inhibitors.

## 4. Monotherapy with PD-1/PD-L1 Inhibitors

Atezolizumab monotherapy was evaluated in the clinical phase I trial PCD4989g for anti-tumor efficacy and safety in advanced or metastatic solid and hematological tumors. The results showed that among 116 evaluable patients, treatment-related adverse events (trAEs) occurred in 73 (63%), and most of them (79%) were grade 1 to 2, which was similar to the other antineoplastic drugs. Patients with mTNBC treated with atezolizumab as a first-line therapy had an objective response rate (ORR) of 24%, and a median overall survival (mOS) of 17.6 months (95% CI:10.2–N/A), and the incidence of trAEs was 62%. In contrast, women treated with atezolizumab as second- or third-line therapy had an ORR of 6% and an mOS of 7.3 months (95% CI: 6.1–10.8). In addition, the study showed that atezolizumab monotherapy had a higher ORR, mOS, and median progression-free survival (mPFS) in patients with mTNBC with higher levels of TILs. This study leads to the preliminary conclusion that first-line treatment with atezolizumab monotherapy is well tolerated and beneficial in patients with advanced TNBC or mTNBC, especially in those with higher levels of TILs [35].

A small-sample phase Ib clinical trial, KEYNOTE-012, is being conducted to determine the safety and anti-tumor activity of pembrolizumab monotherapy in advanced PD-L1-positive mTNBC. All patients included in the study received other prior therapies (i.e., pembrolizumab monotherapy was not used as the first-line therapy). The results showed that among the 27 study subjects with evaluable efficacy, ORR was 18.5% (95% CI: 6.3%–38.1%), including one complete remission and four partial remissions, with a disease-control rate of 25.9% (95% CI: 11.1–46.3%), and mPFS and mOS were 1.9 (95% CI: 1.3–4.3) and 10.2 (95% CI: 5.3–N/A) months. The most common trAEs were arthralgia, fatigue, myalgia, and nausea, with only 15.6% of grade 3–5 trAEs occurring. This result is comparable to the treatment effect of pembrolizumab in other high-grade malignancies [36,37,38]. In addition, this result is similar to the results of second- and third-line treatments in PCD4989g, further demonstrating the authenticity and reliability of both trials.

Pembrolizumab monotherapy in mTNBC was also studied in a clinical phase II trial (KEYNOTE-086). The results showed that patients with PD-L1-positive mTNBC treated with first-line pembrolizumab monotherapy had an mPFS of 2.1 months (95% CI: 1.9–2.0), an mOS of 18 months (95% CI: 12.9–23.0), and an ORR of 21.4%. In contrast, patients with mTNBC who had received prior chemotherapy (i.e., pembrolizumab alone, not as first-line therapy) had an mPFS of 2.0 months (95% CI: 1.9–2.0), an mOS of 9 months (95% CI: 7.6–11.2), and an ORR of only 5.3%. The incidence of trAEs was 63.1% for the first-line treatment population and 60.6% for those who had received other prior treatments, both of which were comparable. This study concluded that pembrolizumab monotherapy has durable anti-tumor activity in patients with PD-L1-positive mTNBC [39]. It further confirmed the effectiveness of PD-1 inhibitors in the treatment of TNBC.

A phase III randomized controlled trial, KEYNOTE-119, compared the efficacy of pembrolizumab monotherapy as non-first-line therapy with chemotherapy for the treatment of mTNBC. The study showed an ORR of 26% for pembrolizumab monotherapy and 12% for chemotherapy in patients with PD-L1 positive tumors and combined positive score (cps) ≥20. Among patients with cps ≥ 10, the mOS was 12.7 months (95% CI: 9.9–16.3), and ORR was 18% for pembrolizumab monotherapy; mOS was 11.6 months (95% CI: 8.3–13.7), and ORR was 9% for chemotherapy. Among patients with cps ≥1, mOS was 10.7 months (95% CI: 9.3–12.5) and ORR was 12% for pembrolizumab monotherapy; the mOS was 10.2 months (95% CI: 7.9–12.6), and ORR was 9% for chemotherapy. Overall, the mOS was 9.9 months (95% CI: 8.3–11.4) for pembrolizumab monotherapy and 10.8 months (95% CI: 9.1–12.6) for chemotherapy. In addition, the incidence of adverse events was comparable between the trAEs of both therapies, except for a statistically significant difference in the incidence of immune-related adverse events [40]. It is evident that pembrolizumab monotherapy did not significantly improve ORR or OS in patients with mTNBC who had previously received other treatments compared to monotherapy. However, as PD-L1 increased in the tumor microenvironment, pembrolizumab monotherapy was more effective, while there was little difference in the efficacy of chemotherapy, suggesting that the degree of clinical benefit of pembrolizumab treatment in patients with mTNBC may be correlated with tumor PD-L1 expression.

In addition, the efficacy of avelumab in monotherapy of locally advanced or metastatic breast cancer was studied in a phase 1 JAVELIN Solid Tumor trial (NCT01772004) [41], demonstrating an acceptable safety profile and clinical activity. However, in-depth studies for Avelumab, such as NCT04360941, NCT03971409, and NCT03971409, are still in progress.

Table 4 summarizes the clinical trial results of monotherapy with PD-1/PD-L1 inhibitors.

By analyzing the results of these trials, we can preliminarily conclude that the application of PD-1/PD-L1 inhibitors for the treatment of locally advanced TNBC or mTNBC has a certain clinical efficacy. Comparing the clinical efficacy with treatment-related adverse reactions shows that the safety of this regimen is guaranteed. Therefore, this regimen could be clinically useful. However, further research is needed to clarify the conditions under which PD-1/PD-L1 inhibitor therapy is indicated and to determine whether there is any clinical benefit compared to chemotherapy, which was the gold-standard treatment in the past. From these trials, we can see that the more positive PD-L1 and high cps, the earlier the application of the treatment, and the better the treatment outcome for patients with advanced TNBC or mTNBC. In addition, the study subjects of the above trial had strict inclusion criteria, their general condition was good, and the actual situation, such as patients′ willingness and economic status, was not considered, so their representativeness was poor. In summary, single-agent immunotherapy has major clinical limitations, and the treatment of mTNBC remains unclear. Therefore, the combination of immune checkpoint inhibitors with other therapies is a noteworthy treatment strategy. 

## 5. Combination Therapy with PD-1/PD-L1 Inhibitors

### 5.1. Combination with Chemotherapy Drugs

A multicenter, randomized, double-blind phase III clinical trial, IMpassion130, evaluated the efficacy and safety of atezolizumab in combination with nab-paclitaxel (trial arm) compared with placebo in combination with nab-paclitaxel (control arm) as a first-line treatment of patients with locally advanced TNBC or mTNBC. The results showed that in the intention-to-treat population (ITT), mPFS was significantly longer in the trial arm than in the control arm, at 7.2 months compared with 5.5 months in the control arm (*p* = 0.0025). This difference was even more significant in PD-L1-positive patients, with an mPFS of 7.5 months and 5.0 months in the two groups, respectively (*p* < 0.0001). In the OS analysis of ITT, mOS was 21.0 months (95% CI:19.0–22.6) in the trial arm compared with 18.7 months (95% CI: 16.9–20.3) in the control arm (stratified hazard ratio (HR) = 0.86, 95% CI: 0.72–1.02, *p* = 0.078); in patients with PD-L1-positive tumors, mOS was 25.0 months (95% CI: 19.6–30.7) in the trial arm versus 18.0 months (95% CI: 13.6–20.1) in the control arm (HR = 0.71, 95% CI: 0.54–0.94). These results suggest that patients with PD-L1-positive tumors benefit more from the atezolizumab plus nab-paclitaxel regimen, which has a manageable safety profile and could be an important option for treating patients with PD-L1-positive mTNBC. Therefore, atezolizumab in combination with an nab-paclitaxel regimen was approved by the FDA in March 2019 for the treatment of locally advanced or metastatic PD-L1-positive TNBC [42]. 

The IMpassion131 study is a double-blind, randomized, phase III clinical trial that evaluated the efficacy and safety of atezolizumab in combination with paclitaxel as a first-line treatment for unresectable locally advanced TNBC or mTNBC. The study design was essentially similar to that of the IMpassion130 study, with the main difference being the chemotherapeutic agents applied. The IMpassion130 study used nab-paclitaxel and the IMpassion130 study used paclitaxel. Unlike the IMpassion130 study, the IMpassion131 study failed to achieve the expected results: in the PD-L1-positive population, the mPFS for Atezolizumab combined with paclitaxel (trial arm) was 6.0 months, compared to 5.7 months for placebo combined with paclitaxel (control arm) (HR = 0.82, 95% CI: 0.60–1.12, *p* = 0.20). In terms of OS, the mOS was 22.1 months in the trial arm compared to 28.3 months in the control arm, and an analysis of the final OS results showed no difference between the two groups (HR = 1.11, 95% CI: 0.76–1.64). The results in the ITT population were consistent with those in the PD-L1-positive population, and the safety profile was consistent with the known effects. Thus, we can conclude that atezolizumab combined with paclitaxel treatment failed to significantly improve the PFS compared to paclitaxel chemotherapy alone. Moreover, the mOS results favored the placebo combined with the paclitaxel regimen in both the PD-L1-positive population and the total population [43]. Therefore, recently, Atezolizumab, in combination with chemotherapy, is no longer FDA-approved for the treatment of patients with advanced TNBC whose tumors express PD-L1 (Immune Cell score ≥ 1%), yet holding this indication according to the European Medicines Agency (EMA).

A multicenter, prospective, randomized, double-blind, placebo-controlled phase 2 trial GeparNuevo enrolled 174 patients with primary cT1b-cT4a-d disease, centrally confirmed TNBC and sTILs. Patients were divided into two groups and given durvalumab or placebo every 4 weeks in addition to nab-paclitaxel followed by standard EC. In the window phase, durvalumab/placebo alone was given 2 weeks before start of nab-paclitaxel. In this study, 53.4% achieved a pCR (ypT0 ypN0) treated with durvalumab, compared with 44.2% treated with placebo. Unfortunately, this result did not reach statistical significance. However, durvalumab effect was seen in the window cohort, and the pCR rate was increased by treating with durvalumab alone before start of chemotherapy (61.0% versus 41.4%, OR = 2.22, 95% CI 1.06–4.64, *p* = 0.035; interaction *p* = 0.048) [44]. These results suggest that combining immune checkpoint inhibitors with chemotherapy may improve response rates in patients with mTNBC.

In an open-label, single-arm trial (NCT02628132), 14 patients received five cycles of weekly paclitaxel concurrently with biweekly durvalumab. In this trial, the combination of durvalumab and paclitaxel had an mPFS of 4.0–5.0 months, which was not significantly different from using paclitaxel alone (3.5–5.3 months). Although the OS at 20.7 months was higher than that of paclitaxel monotherapy, the study’s sample size was too small to have a control arm, so it could only be compared to previous studies [45]. This obviously impairs the strength of the conclusions. Large prospective randomized trials are needed in the future to further determine the effectiveness of this treatment.

The KEYNOTE-355 study is a double-blind, randomized, multicenter phase III clinical trial designed to evaluate the efficacy and safety of pembrolizumab in combination with chemotherapy (albumin, paclitaxel, gemcitabine, and carboplatin) compared with placebo in combination with chemotherapy for unresectable locally advanced TNBC or mTNBC. The study was stratified by the type of chemotherapy (paclitaxel-based agents or gemcitabine and carboplatin), PD-L1 expression at baseline (cps), and whether the previous chemotherapy category was the same in neoadjuvant or adjuvant therapy. The results showed that for those with PD-L1 positivity and cps ≥ 10, mPFS was 9.7 months for pembrolizumab combined with chemotherapy (trial arm) and 5.6 months for placebo combined with chemotherapy (control arm) (HR = 0.65, 95% CI: 0.49–0.86, one-sided *p* = 0.0012). Among those with cps ≥1, mPFS was 7.6 months in the trial arm and 5.6 months in the control (HR = 0.74, 95% CI:0.61–0.90, one-sided *p* = 0.0014). In addition, subgroup analysis revealed that the clinical efficacy of pembrolizumab combined with chemotherapy had the most significant benefit in those with cps ≥ 20, followed by those with cps ≥10, and those with cps ≥1. This further confirms that the therapeutic effect of PD-1 inhibitors in combination with chemotherapy increases with the enrichment of PD-L1. An analysis of adverse drug reactions showed that the pembrolizumab combination chemotherapy regimen was well tolerated by the patients, and no new safety issues were identified [46]. These findings suggest that combining pembrolizumab with standard chemotherapy is effective as a first-line treatment for mTNBC.

The KEYNOTE-522 study is another phase III study that was also designed to evaluate the efficacy of pembrolizumab in combination with chemotherapy compared with placebo in combination with chemotherapy for mTNBC. Data showed that during the treatment period, regardless of PD-L1 expression levels, the pCR of patients with a regimen of pembrolizumab in combination with chemotherapy was 64.8% (95% CI: 59.9–69.5), which was significantly higher than that of 51.2% (95% CI: 44.1–58.3) with chemotherapy only. The above studies suggest that combining pembrolizumab with standard chemotherapy is effective as a first-line treatment for mTNBC. Additionally, pembrolizumab, in combination with chemotherapy, has been approved by the FDA for patients with high-risk early TNBC as a (neo)adjuvant treatment, and for first-line treatment of patients with advanced TNBC whose tumors express PD-L1 (cps ≥ 10).

The immune combination protocol evaluated the efficacy of PD-1/PD-L1 inhibitors in combination with “gold standard” chemotherapy regimens compared with standardized chemotherapy regimens alone. The results of these trials differed for different chemotherapeutic agents. This suggests that there is large heterogeneity among patients with TNBC and that immunotherapy in combination with chemotherapy still faces many challenges, and it is worthwhile to further explore how to choose the best combination partner and the population that benefits from immunotherapy. Therefore, after a comparative analysis of the two trials, IMpassion130 and IMpassion131, the IMpassion132 study was conducted. This study expands on the two trials mentioned above to include patients with TNBC who had relapsed within 12 months after early chemotherapy and to evaluate the efficacy and safety of atezolizumab in combination with paclitaxel and nab-paclitaxel treatment, respectively, to further explore the optimal population for immunosuppressive therapy. The project is still in progress, and results are expected in March 2024 [47].

The NCT04085276 study is a phase III, multicenter, randomized, double-blind study, which aims to evaluate the efficacy and safety of Toripalimab (JS001) combined with nab-paclitaxel compared with placebo combined with nab-paclitaxel for first/second line treatment of metastatic or recurrent TNBC. This study will evaluate PFS, ORR, duration of response (DOR), disease control rate (DCR) and OS. No relevant results have been reported by the investigators.

A phase 2 TONIC trial investigated the efficacy of nivolumab administered after induction with different regimens of irradiation, cyclophosphamide, cisplatin, and doxorubicin in metastatic TNBC. Overall, the ORR was 20%, with most responses occurring in the cisplatin (ORR 23%) and doxorubicin (ORR 35%) cohorts [48]. The study suggests that after doxorubicin and cisplatin induction, a favorable immune microenvironment will develop and PD-1 will be more easily blocked. The results of this trial demonstrate the great potential of nivolumab in combination with chemotherapeutic agents, but more data are pending to support this conclusion due to the small number of clinical trials designed with nivolumab.

Table 5 summarizes the clinical trial results of PD-1/PD-L1 inhibitors in combination with chemotherapy.

### 5.2. Combination with Small Molecule Inhibitors

Poly ADP-ribose polymerase (PARP) is a DNA damage-repair protein that repairs DNA damage by binding to DNA damage sites and catalyzing the synthesis of poly ADP-ribose chains on protein substrates. A cell-killing mechanism called “synthetic lethality” exists in the body for its own abnormal cells. A synthetic lethal interaction occurs between two genes when a perturbation (a mutation, RNA interference knockdown, or inhibition) that affects either gene alone is viable but the perturbation of both genes simultaneously is lethal. Due to the presence of damage-repair mechanisms within the cell, this “synthetic lethality” does not normally occur. However, when the damage-repair mechanism is impaired, it leads to the accumulation of perturbations that induce the death of these cells [49]. By binding to the PARP catalytic site, the PARP inhibitor prevents the PARP protein from being shed from the DNA damage site, which leads to DNA replication fork stalling and DNA replication not proceeding smoothly. When the cell normally triggers homologous recombination repair (HRR), a complex signaling pathway involving multiple steps, the most critical proteins are BRCA1 and BRCA2 [50]; thus, cancer patients carrying mutations in the BRCA1 or BRCA2 germline have concurrent HRR malfunctions in their bodies, at which point the cells, in turn, employ other DNA repair methods. However, other DNA repair methods usually introduce massive genomic reorganization, resulting in the simultaneous presence of two or more gene or protein abnormalities in the cell, which triggers cell death. It is important to note that while PARP inhibitors are commonly associated with tumors exhibiting mutations in the BRCA1 or BRCA2 germline, they may also be effective against other types of tumors. This is due to the fact that many other types of tumor cells do not have BRCA1/2 germline mutations, but instead have other intracellular causes mediating HRR defects, resulting in the sensitivity of these tumor cells to PARP inhibitors [49]. PARP inhibitors have been successively approved by the FDA for the treatment of ovarian cancer, fallopian tube cancer, and peritoneum. In 2018, the FDA approved PARP inhibitor monotherapy for the treatment of HER2-negative metastatic breast cancer caused by deleterious germline BRCA mutations. However, the combination regimen of PARP inhibitors and immunotherapy is still in trials.

A phase I/II clinical trial, KEYNOTE-162, was designed to evaluate the safety and efficacy of the PARP inhibitor niraparib in combination with pembrolizumab for the treatment of patients with unresectable locally advanced TNBC or mTNBC. The results showed that of 45 patients with assessable overall efficacy, 13 (29%) achieved complete or partial objective remission, and patients with PD-L1-positive tumors responded better than those with PD-L1-negative tumors (33% vs. 8%). Regardless of the BRCA mutation, the regimen of niraparib combined with pembrolizumab showed potential anti-tumor activity in patients with advanced TNBC or mTNBC. The trAEs above grade 3 were mainly anemia, thrombocytopenia, and fatigue, indicating a good safety profile of the therapy [51], and are ready for the next phase of clinical trials.

Upregulation of mitogen-activated protein kinase (MAPK) expression is often a marker of cancer development and progression. Multiple signaling molecules in cells bind to tyrosine receptors and activate RAS proteins, which in turn phosphorylate Raf and activate MEK (MEK1 and MEK2) and its substrates ERK (ERK1 and ERK2), according to cascade signaling. Ultimately, ERK acts on different downstream molecules to regulate a series of key cellular activities, such as cell proliferation, invasion, angiogenesis, and apoptosis resistance. Therefore, in tumor cells, mutations in either K-ras or B-raf upstream of the MAPK pathway lead to the abnormal activation of ERK, allowing tumor cells to develop. However, since different cell lines have different mutation sites, the clinical efficacy of single RAS or RAF inhibitors is limited, whereas MEK inhibitors have significant efficacy in malignancies caused by either K-ras or B-raf mutations [52].

The COLET trial is a randomized, multicenter, three-cohort, phase II study that explored the efficacy and safety of a three-drug regimen of the MEK inhibitor cobimetinib in combination with atezolizumab and chemotherapy as the first-line treatment of patients with locally advanced TNBC or mTNBC. The results showed that the ORR was 38.3% (95% CI: 24.40–52.20%) for cobimetinib in combination with paclitaxel and 20.9% (95% CI: 8.77–33.09%) for placebo in combination with paclitaxel. The ORR of cobimetinib combined with atezolizumab and paclitaxel was 34.4% (95% CI: 18.57–53.19%), and the mPFS was 3.8 months; the ORR of cobimetinib combined with atezolizumab and nab-paclitaxel was 29.0% (95% CI: 14.22–48.04%), and the mPFS was 7.0 months. The analysis revealed that only a non-significant increasing trend in PFS or ORR was observed with the combination of MEK inhibitors on top of paclitaxel. MEK inhibitors combined with PD-1 inhibitors and paclitaxel did not achieve better clinical outcomes compared to those combined with paclitaxel alone [53]. Therefore, further research is needed regarding MEK inhibitor-related therapies.

Extracellular-5′-nucleotidase (CD73) is a major enzyme located on the cell surface encoded by the NT5E gene, which catalyzes the formation of extracellular adenosine from AMP and coordinates the homeostatic balance of extracellular adenosine levels [54]. It is present on the surface of cells such as endothelial cells, lymphocytes, stromal cells, and tumor cells. Adenosine is a potent immunosuppressive molecule that inhibits T cell proliferation, cytotoxicity, and cytokine production. Adenosine also promotes the proliferation of regulatory T cells and stimulates myeloid-derived suppressor cell (MDSC) and macrophage M2 polarization, thereby exerting an immunosuppressive effect. In addition, adenosine can promote proliferation, angiogenesis, and metastasis of cancer cells [55]. The expression of CD73 and the release of adenosine in tumor cells are closely related to tumor invasion and metastasis [56], which leads to the dysregulation of CD73 expression in breast cancer, metastatic melanoma, and ovarian cancer [57]. Furthermore, the overexpression of CD73 in tumors not only leads to dysregulation of adenosine production, which in turn leads to immune escape and promotes tumor metastasis, but also leads to tumor resistance to anthracyclines. Therefore, some studies have combined CD73 inhibitors with PD-1/PD-L1 inhibitor therapies for the treatment of cancer.

A multicenter, randomized, open phase II clinical trial, the SYNERGY trial (NCT03616886), was designed to evaluate the efficacy and safety of immunotherapy (durvalumab + MEDI9447 [CD73 inhibitor]) in combination with chemotherapy (paclitaxel and carboplatin) as the first-line treatment for unresectable locally advanced TNBC or mTNBC. However, this study is currently in the trial phase and is expected to be completed by 2023.

The FDA has approved CTLA-4 inhibitors for the treatment of advanced melanoma that cannot be surgically treated. Therefore, a single-arm, phase II study (NCT0253679) evaluated the efficacy and safety of the CTLA-4 inhibitor, tremelimumab, in combination with durvalumab in patients with mTNBC. A total of seven patients with mTNBC were recruited into the trial, of whom three achieved an overall remission rate of 43%, and 71% of them achieved clinical benefit. They were all patients with TNBC; however, as the damage from immune-related adverse reactions that occurred in this study outweighed the benefits, indicating that the safety of the regimen could not be guaranteed, it did not progress to the second phase of the study [58].

There are also a number of small molecules that play an important role in the progression of breast cancer. For example, vascular endothelial growth factor (VEGF) and vascular endothelial growth factor receptor (VEGFR) play a key role in the angiogenesis of breast cancer [59]; platelet-derived growth factor (PDGF) is expressed at a high frequency in invasive breast cancer [60]; the co-expression of stem cell factor and c-kit leads to dysregulation of breast cancer growth [61]. Famitinib is a tyrosine kinase inhibitor that targets c-kit, VEGFR-2, VEGFR-3, PDGFR, and other receptor tyrosine kinases [62].

An open-label, single-arm, phase II study, NCT04129996, enrolled patients with previously untreated, advanced, immunomodulatory TNBC. In this study, the ORR was very high, reaching 81.3% (95% CI: 70.2–92.3), with 5 complete and 34 partial responses. The mPFS was 13.6 months (95% CI: 8.4–18.8), and the median duration of response (DOR) was 14.9 months. No treatment-related deaths were reported in this study, suggesting that the triple combination of Famitinib with Camrelizumab and Nab-Paclitaxel is highly efficacious and well-tolerated in previously untreated advanced immune tumors [63]. However, this study did not have a control arm and has certain design flaws, and the benefits of its efficacy still need to be confirmed by further studies. In addition, a variety of treatments are still in clinical trials, including IL-8/CXCR inhibitors, breast cancer vaccines, and lysing viruses, angiogenesis inhibitors, and inhibitors of cyclin-dependent kinase 4 (CDK4) and CDK6, in combination with PD-1/PD-L1 inhibitors [64,65].

Table 6 summarizes the clinical trial results of PD-1/PD-L1 inhibitors in combination with small molecule inhibitors.

From the above trials, it is clear that different treatment regimens have different effects on the therapeutic effect and prognosis of mTNBC. Therefore, the treatment of mTNBC should take factors such as the tumor load, molecular characteristics, recurrence and metastasis pattern, previous treatment, patient’s performance status into account, in order to classify TNBC into multiple types to facilitate precise treatment. Nowadays, a recommended standard of care for TNBC has been developed internationally (Table 7) [66].

## 6. New Targets for Immunotherapy

Protein tyrosine phosphatase non-receptor type 2 (PTPN2) is a member of the protein tyrosine phosphatase (PTP) family, which is involved in regulating cell growth, differentiation, division, and oncogenic transformation. Numerous studies have shown that the knockdown of the PTPN2 gene in mouse T cells not only promotes T-cell expansion and conversion of progenitor T cells to cytotoxic T cells but also promotes the release of granzyme B from cytotoxic T cells, enhancing the ability to kill tumor cells [67]. A study in 2019 showed that PTPN2 induced CD8+T cell subpopulation depletion acted synergistically with PD-1-mediated immunosuppressive responses. The knockdown of PTPN2 in the immune system of mice resulted in the complete elimination of colon cancer foci in mice bearing colon cancer and, in combination with PD-1 inhibitors, resulted in the elimination of approximately 1/4 of tumor foci in mice bearing highly aggressive and treatment-resistant melanoma. In contrast, treatment with PD-1 inhibitors alone failed to eliminate tumors in one of the tumor-bearing mice [68]. Therefore, dual inhibition therapy with PTPN2 and PD-1/PD-L1 is one of the most promising cancer treatments available [69].

The IL-33/ST2 pathway promotes tumor progression by suppressing anti-tumor immunity and promoting angiogenesis. IL-33 promotes the expression of immunosuppressive molecules such as PD-1 by CD8+T cells, which depletes T cells [70]. Studies have shown that the inhibition of the IL-33/ST2 pathway enhances anti-tumor immunity and slows down the progression of tumors [71,72]. Combined blockade of the IL33/ST2 and PD-1/PD-L1 pathways can promote the accumulation of CD4+ and CD8+ T lymphocytes [73], enhance the activity of NK cells, and have better anti-tumor efficacy than treatments that block the IL33/ST2 or PD-1/PD-L1 pathway alone (*p* < 0.05) [74], indicating that it is also a potential new approach for immunotherapy.

Leucine-rich repeat protein 33 (LRRC33), which is required for antigen presentation on the surface of tumor-infiltrating bone marrow cells, binds specifically to transforming growth factor 1 (TGF-β1, a key factor that regulates wound healing, immune response, and tumor development [75]) and activates the TGF-β1 signaling pathway. In a mouse model, blocking the LRRC33/TGF-β1 pathway resulted in a reduction in myeloid suppressor cells in the immunological microenvironment and enhanced the activity of CD8^+^ T and NK cells and the polarization of macrophages toward M1, thereby slowing tumor growth and metastasis [76]. In addition, TGF-β1 inhibitors can inhibit the PD-1/PD-L1 pathway and attenuate its negative regulatory effects on anti-tumor immunity [77,78,79]. In a mouse model where anti-PD-1 treatment was ineffective, the combined use of SRK-181-mIgG1 (specifically inhibiting TGF-β1) and anti-PD-1 antibody resulted in an increase in intra-tumor CD8^+^ T cells and a decrease in immunosuppressive myeloid cells, indicating an anti-tumor effect [80]. The above results suggest that the combined blockade of the LRRC33/TGF-β1 and PD-1/PD-L1 signaling axes is potentially feasible for tumor immunotherapy.

Autophagy is a complex intracellular phenomenon that separates and degrades various cytoplasmic structures through lysosomes. The activation of autophagy can improve the body’s immune surveillance of tumors while increasing tumor antigenicity and further activating the body’s anti-tumor immune response. Insulin-like growth factor 1 receptor (IGF1R) is one of the most important trophic receptor tyrosine kinases, stimulating the uptake of nutrients into cells as well as a variety of anabolic reactions. The inhibition of IGF1R itself or that of the signal transduction cascade acting downstream of IGF1R (the PI3K/AKT/MTOR pathway) potently stimulates autophagy. The IGF1R inhibitor activates the autophagic process, which not only improves the body’s immune surveillance but also forces tumors to release adenosine triphosphate (ATP), an immunogenic marker, further enhancing the body’s anti-tumor immune response. Experiments have shown that IGF1R inhibitors can enhance the efficacy of anticancer chemotherapies, alone or in combination with PD-1-blocking antibodies in mouse models. At the clinical level, this study has also observed that the activating phosphorylation of IGF1R detectable by immunohistochemistry is correlated with poor immunosurveillance and disease control in TNBC patients. Therefore, IGF1R inhibitors will provide new options for immunotherapy of TNBC [81].

Cancer testis antigens (CTAs) are a family of multifunctional proteins that are specifically expressed in male spermatozoa and tumor cells but not in healthy somatic cells. CTAs are not only closely related to the stemness of tumor cells, tumorigenicity, mobility, metastasis, and the drug resistance of cancer cells, but they also show high tumor specificity and sensitivity. CTAs are immunogenic proteins, so they can trigger cellular immunity and humoral immunity. Animal experiments have shown that the combined application of CTAs antibodies with PD-1/PD-L1 inhibitors has a significant killing effect on tumor cells. Kita-Kyushu lung cancer antigen-1 (KK-LC-1, also known as CT83 or cxorf61) is a CTA and is highly expressed in lung cancer, gastric cancer, and breast cancer. Therefore, KK-LC-1 is a new target for immunotherapy and may become a valuable tumor-related marker in the future. Although there is currently no literature reporting on the combination of KK-LC-1 and immune checkpoint inhibitors, the simultaneous use of both may be a valuable method for clinical applications in the future [82].

## 7. Adverse Events Associated with PD-1/PD-L1 Inhibitors

With the development of new drugs, more attention has been paid to the safety of drugs. Adverse events are an important indicator to evaluate drug safety. Common treatment-related adverse events include arthralgia, asthenia, anemia [83], neutropenia, and peripheral neuropathy [84]. Common immune-related adverse events (irAEs) include thyroid diseases (hypothyroidism [85], hyperthyroidism, thyroiditis, etc.), adrenal insufficiency, diabetes, dermal toxicity, autoimmune hepatitis gastrointestinal toxicity, and pneumonia. Other rare adverse events have also been reported [86].

A meta-analysis included clinical data from six studies involving 586 patients with advanced breast cancer who demonstrated a controlled safety profile after monotherapy with pembrolizumab, atezolizumab, or avelumab. The results showed that adverse events occurred in more than 64% of patients, the incidence of severe adverse events and irAEs was about 13% and 15%, about 3% of patients stopped treatment because they could not tolerate adverse events, and 0.31% of patients died from severe treatment-related AEs [83].

Another systematic review, which included 3007 patients from four studies, showed more adverse events with immunotherapy plus chemotherapy compared to chemotherapy [87]. Some scholars similarly concluded that the combination of immune checkpoint inhibitors (ICI) with neoadjuvant chemotherapy (NACT) was more likely to lead to grade 3/4 AEs (OR 1.31, *p* = 0.02) and severe AEs (OR 1.84, *p* = 0.006) [88].

Although PD-1/PD-L1 inhibitors play an important role in the treatment of mTNBC, the accompanying adverse events still deserve attention. Most of the adverse events were released along with the study results, which lacked systematic statistics and analysis, making it difficult to ensure that the results would not be affected by subjective factors of each research center. In addition, the efficacy and safety of the same treatment may vary from patient to patient. Therefore, it is necessary to conduct a large-scale statistical study to further evaluate the safety of PD-1/PD-L1 inhibitor therapy.

## 8. Conclusions

TNBC is known for its extremely high drug resistance and progressiveness, poor prognosis, and lack of ease of treatment in all types of breast cancer. To address these challenges, immunotherapy is a revolutionary breakthrough in the process of fighting and defeating cancer. The development of immune checkpoint inhibitors and their increasing clinical use has ushered in a new era of immunotherapy for mTNBC. While there are high expectations for immunotherapy, it is also important to recognize that there are still many issues related to immunotherapy, such as adverse effects, which have yet to be addressed. We should achieve a deeper understanding of the interaction between tumors and the immune system and strive to explore more effective immunotherapy regimens for mTNBC so that patients can receive better treatment.

## Figures and Tables

**Table 1:** Common immunotherapy approaches.

Common Immunotherapy Approaches	Principle	Current Clinical Applications
Immune checkpoint blockade therapy	This is a type of therapy that blocks the action of immune checkpoints by artificially administering inhibitors of immune checkpoints or their ligands, thereby upregulating T cells activity and improving the body’s anti-tumor immune response. The most commonly used inhibitors are monoclonal antibodies to the corresponding molecules, such as PD-1/PD-L1 monoclonal antibodies and CTLA-4 monoclonal antibodies.	breast cancer, lung cancer, hepatocellular carcinoma, prostate cancer, melanoma, MSI-H/dMMR colorectal cancer RCC, lymphoma, MCC, urothelial cancer [18].
Therapeutic antibodies	Laboratory-designed antibodies destroy tumor cells by inducing direct apoptosis, antibody-dependent cytotoxicity, and complement-dependent cytotoxicity. Common therapeutic antibodies include rituximab and panitumumab.	breast cancer, colorectal cancer, lymphoma, melanoma, head and neck cancer, NSCLC, RCC, cervical cancer [19].
Cancer vaccine	Tumor antigens are introduced into patients in the form of tumor cells, tumor-related proteins or peptides, and genes that express tumor antigens, so as to activate patients′ own immune responses and reduce immune suppression caused by tumors, thus achieving control or clearance of the tumor. They can be divided into prophylactic and therapeutic vaccines, such as the cervical cancer vaccine and the Sipuleucel-T vaccine.	pancreatic cancer, lymphoma, breast cancer, NSCLC, gastric cancer, glioblastoma, cervical cancer, prostate cancer [20].
Adoptive cellular immunotherapy	Immune cells are collected from the patient’s blood, and the collected immune cells are then genetically edited to change ordinary immune cells into immune cells that can recognize tumor cells, expanded and cultured, and then infused back into the patient with such immune cells that can trigger the killing effect of tumor cells, thus playing the role of anti-tumor immunity. The available immune cells are autologous lymphokine-activated killer cells, natural killer cells, cytokine-induced killer cells, cytotoxic T cells, and genetically modified T cells, etc.	melanoma, renal cell carcinoma, breast cancer, cervical cancer, gastrointestinal cancers, cholangiocarcinoma, pancreatic cancer, head and neck cancer, ovarian cancer, NSCLC [21].
Small-molecule inhibitors	There are many small-molecule proteins in tumor cells and in the tumor microenvironment, which can promote the occurrence and development of tumors by inhibiting the anti-tumor immunity, and promoting the accumulation of abnormal mutations and the abnormal proliferation of tumor cells. By artificially providing inhibitors of these small-molecule proteins, the above abnormal responses can be cut off and tumor progression can be inhibited. Common small-molecule inhibitors include IDO inhibitors, PARP inhibitors, MEK inhibitors, VEGFR inhibitors, etc.	breast cancer, ovarian cancer, thyroid cancer, soft tissue sarcoma, colorectal cancer, melanoma, pancreatic cancer, renal cell carcinoma, NSCLC, leukemia [22].

Abbreviations: CTLA-4, cytotoxic T lymphocyte-associated antigen-4; IDO, indoleamine 2,3-dioxygenase; PARP, poly ADP-ribose polymerase; VEGFR, vascular endothelial growth factor receptor; NSCLC, non-small lung cancer; MSI-H, high levels of Microsatellite Instability; dMMR, different mismatch repair; HCC, hepatocellular carcinoma; RCC, renal cell carcinoma; MCC, Merkel cell carcinoma.

**Table 2:** PD-1 inhibitors currently in use for clinical treatment.

Generic Name	Approved for	R&D Company	Degree of Antibody Humanization	Antibody Type
Nivolumab	NSCLC, head and neck squamous cell carcinoma, pleural mesothelioma, gastroesophageal junction carcinoma, gastric cancer, melanoma.	Bristol-Myers Squibb Pharm EEIG (New York, NY, the US)	Fully human	IgG4
Pembrolizumab	Melanoma, Hodgkin’s lymphoma, NSCLC, head and neck squamous cell carcinoma, esophageal cancer, advanced MSI-H/dMMR colorectal carcinoma.	Merck Sharp & Dohme Corp (Beijing, China)	Humanized	IgG4k
Camrelizumab	Non-squamous NSCLC, classical Hodgkin’s lymphoma, nasopharyngeal carcinoma, HCC, esophageal squamous carcinoma.	Suzhou Shengdiya Biopharmaceutical Co. (Suzhou, China)	Humanized	IgG4k
Toripalimab	Melanoma, nasopharyngeal carcinoma, uroepithelial carcinoma, esophageal squamous carcinoma.	Shanghai Junshi Biomedical Technology Co. (Shanghai, China)	Humanized	IgG4k
Tislelizumab	(Non-)squamous NSCLC, hepatocellular carcinoma, Hodgkin’s lymphoma, uroepithelial carcinoma.	Baekje Shenzhou (Shanghai) Biotechnology Co. (Shanghai, China)	Humanized	IgG4
Penpulimab	Hodgkin’s lymphoma.	Zhongshan Kangfang Bio-pharmaceutical Co. (Zhongshan, China)	Humanized	IgG1
Sinitilimab	Squamous lung cancer, non-squamous NSCLC, HCC, Hodgkin’s lymphoma.	Cinda Biopharma (Suzhou) Co. (Suzhou, China)	Fully human	IgG4
Zimberelimab	Hodgkin’s lymphoma.	Guangzhou Yu Heng Biotechnology Co. (Guangzhou, China)	Fully human	IgG4

Abbreviations: NSCLC, non-small lung cancer; MSI-H, high levels of microsatellite instability; dMMR, different mismatch repair; HCC, hepatocellular carcinoma.

**Table 3:** PD-L1 inhibitors currently in use for clinical treatment.

Generic Name	Approved for	R&D Company	Degree of Antibody Humanization	Antibody Type
Atezolizumab	Breast cancer, uroepithelial cancer, (non-) small cell lung cancer, HCC.	Genentech (Roche) (San Francisco, the US)	Humanized	IgG1k
Durvalumab	(Non-) small cell lung cancer.	AstraZeneca (London, the UK)	Fully human	IgG1k
Avelumab	Metastatic MCC, uroepithelial carcinoma.	EMD Serono (Merck/Pfizer) (Darmstadt, Germany)	Fully human	IgG1

Abbreviations: HCC, hepatocellular carcinoma; MCC, Merkel cell carcinoma.

**Table 4:** Clinical trial results for PD- 1/PD-L1 inhibitor monotherapy.

Test Name	Identifiers	Test Arm	Control Arm
PCD4989g (Phase I)	NCT01375842	ORR: 24% mOS: 17.6 months (95% CI: 10.2–N/A) trAEs: 62%	ORR: 6% mOS: 7.3 months (95% CI: 6.1–10.8) trAEs: 43%
KEYNOTE-01 (Phase Ib)	NCT01848834	ORR: 18.5% (95% CI: 6.3–38.1%) mPFS: 1.9 months (95% CI: 1.3–4.3) mOS: 10.2 months (95% CI: 5.3–N/A) Level 3–5 trAEs: 15.6%	-
KEYNOTE-086 (Phase II)	NCT02447003	ORR: 21.4% mPFS: 2.1 months (95% CI: 1.9–2.0) mOS: 18 months (95% CI: 12.9–23.0) trAEs: 63.1%	ORR: 5.3% mPFS: 2.0 months (95% CI: 1.9–2.0) mOS: 9 months (95% CI: 7.6 –11.2) trAEs: 60.6%
KEYNOTE-119 (Phase III)	NCT02555657	mOS: 9.9 months (95% CI: 8.3–11.4) cps≥20: ORR: 26% cps≥10: ORR: 18% mOS: 12.7 months (95% CI: 9.9–16.3) cps≥1: ORR: 12% mOS: 10.7 months (95% CI: 9.3–12.5)	mOS: 10.8 months (95% CI: 9.1–12.6) ORR: 12% ORR: 9% mOS: 11.6 months (95% CI: 8.3–13.7) ORR: 9% mOS: 10.2 months (95% CI: 7.9–12.6)

Abbreviations: mPFS, median progression free survival; mOS, median overall survival; trAEs, treatment related adverse events; ORR, objective remission rate; cps, combined positive score.

**Table 5:** Clinical trial results for PD- 1/PD-L1 inhibitors in combination with chemotherapy.

Test Name	Identifiers	Test Arm	Control Arm
IMpassion130 (phase III)	NCT02425891	ITT: mPFS: 7.2 months mOS: 21.0 months (95% CI: 19.0–22.6) PD–L1–positive: ORR:53% mPFS: 7.5 months mOS: 25.0 months (95% CI: 19.6–30.7)	mPFS: 5.5 months mOS: 18.7 months (95%CI: 16.9–20.3) ORR:33% mPFS: 5.0 months mOS: 18.0 months (95% CI: 13.6–20.1)
IMpassion131 (phase III)	NCT03125902	mPFS: 6.0 months mOS: 22.1 months	mPFS: 5.7 months mOS: 28.3 months
GeparNuevo (phase II)	-	normal cohort: pCR: 53.4% window cohort: pCR: 61.0%	pCR: 44.2% pCR: 41.4%
-	NCT02628132	mPFS: 4.0–5.0 months	-
KEYNOTE-355 (phase III)	NCT02819518	cps≥10: mPFS: 9.7 months cps≥1: mPFS: 7.6 months	mPFS: 5.6 months mPFS:5.6 months
KEYNOTE-522 (phase III)	NCT03036488	pCR: 64.8% (95% CI: 59.9–69.5)	pCR: 51.2% (95% CI: 44.1–58.3)
TONIC trial (phase II)	-	doxorubicin cohort: ORR: 35% cisplatin cohort: ORR: 23%	-

Abbreviations: ITT, intention-to-treat population; mPFS, median progression free survival; mOS, median overall survival; cps, combined positive score; pCR, pathological complete response; ORR, objective remission rate.

**Table 6:** Clinical trial results for PD-1/PD-L1 inhibitors in combination with small molecule inhibitors.

Test Name	Identifiers	Result	
KEYNOTE-162 (phase I/II)	NCT02657889	ORR: 21% PD-L1-positive: 33% tBRCA mutation: 47% mPFS: tBRCA mutation: 8.3months DCR: 49% tBRCA mutation: 80%	PD-L1-negative: 15% tBRCA wild-type: 11% tBRCA wild-type: 2.1months tBRCA wild-type: 33%
COLET (phase II)	NCT02322814	ORR: C+P: 38.3% (95% CI: 24.40–52.20%) placebo+P: 20.9% (95% CI: 8.77–33.09%) C+A+P: 34.4% (95% CI: 18.57–53.19%) C+A+nab-P: 29.0% (95% CI: 14.22–48.04%) mPFS: C+A+P: 3.8 months C+A+nab-P: 7.0 months	
-	NCT04129996	ORR: 81.3% (95% CI: 70.2–92.3) mPFS: 13.6 months (95% CI: 8.4–18.8) median DOR: 14.9 months	

Abbreviations: mPFS, median progression free survival; ORR, objective remission rate; DCR, disease control rate; DOR, duration of response; C, cobimetinib (MEK inhibitor); A, atezolizumab; P, paclitaxel.

**Table 7:** The current standard of care for TNBC.

	First-Line	Second-Line	Third-Line
Patients sensitive to paclitaxel treatment	1. In early-stage TNBC, the current regimen remains anthracycline- or paclitaxel-based single-agent or combination chemotherapy. 2. For locally advanced or metastatic PD-L1-positive TNBC, the recommended regimen is PD-L1 inhibitors in combination with chemotherapy.	1. single-agent chemotherapy 2. nab-paclitaxel in combination with PD-L1 inhibitors	1 chemotherapeutic drug liposomes 2. PD-L1 inhibitors in combination with chemotherapy.
Patients who have failed paclitaxel therapy	1. In early-stage TNBC, the current regimen remains anthracycline- or paclitaxel-based single-agent or combination chemotherapy. 2. For locally advanced or metastatic PD-L1-positive TNBC, the recommended regimen is PD-L1 inhibitors in combination with chemotherapy.	1. single-agent chemotherapy 2. multi-drug combination chemotherapy	1. chemotherapeutic drug liposomes

## Data Availability

No new data were created or analyzed in this study. Data sharing is not applicable to this article.
